# Supplementary figures and images for: Determining organizational resilience through employee resilient characteristics and supportive HR practices: The moderating effect of managerial resilience
Source: PLoS One. 2025 Nov 7;20(11):e0335751. doi: 10.1371/journal.pone.0335751 (PMC12594392; doi:10.1371/journal.pone.0335751)

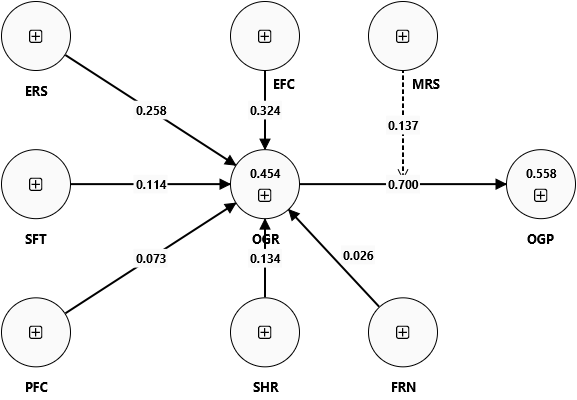

Supplement: S1 Appendix — (DOCX) [file pone.0335751.s005.docx]
