# Supplementary material for: Determining organizational resilience through employee resilient characteristics and supportive HR practices: The moderating effect of managerial resilience
Source: PLoS One. 2025 Nov 7;20(11):e0335751. doi: 10.1371/journal.pone.0335751 (PMC12594392; doi:10.1371/journal.pone.0335751)
Supplement: Attachment — Submitted filename: 2nd Response Letter.docx [file pone.0335751.s009.docx]

**Response Sheet**

Respected reviewer with reference to manuscript ID Number: PONE-D-25-04952R1 “Determining organizational resilience through employee resilient characteristics and supportive HR practices: the moderating effect of managerial resilience” author sincerely appreciate the time and effort the reviewers have dedicated to providing insightful and constructive feedback on our manuscript. Author has carefully addressed all comments to improve the clarity, rigor, and contribution of the manuscript. For detail response please see following response sheet.

| Comments From Reviewer/ Editor | |
| --- | --- |
| Comments | Author Response |
| Authors made some improvements. Change the table format, eliminate the lines inside the table.  Please look at the table format in the Journal articles. |  |

Thank you for your valuable and constructive feedback. We appreciate your thoughtful guidance in strengthening our work.
